# Fitness cost and biofilm formation in fosfomycin-resistant clinical *Escherichia coli* and *Klebsiella pneumoniae* isolates

**DOI:** 10.55730/1300-0144.6165

**Published:** 2025-12-21

**Authors:** Mustafa ÖKEER, Sabire Şöhret AYDEMİR, Bayrı ERAÇ

**Affiliations:** 1 Department of Pharmaceutical Microbiology, Faculty of Pharmacy, Ege University, İzmir, Turkiye; 2 Department of Medical Microbiology, Faculty of Medicine, Ege University, İzmir, Turkiye

**Keywords:** Fosfomycin, fitness cost, virulence, biofilm, *Escherichia coli*, *Klebsiella pneumoniae*

## Abstract

**Background/aim:**

Fosfomycin has regained importance owing to its unique mechanism of action and effectiveness against extended-spectrum β-lactamase-producing Gram-negative bacteria. This study aimed to evaluate the biological fitness cost associated with fosfomycin resistance and its impact on biofilm formation in clinical *Enterobacteriaceae* isolates.

**Materials and methods:**

A total of 78 *Escherichia coli* and 34 *Klebsiella pneumoniae* strains isolated from urine samples at Ege University Hospital were analyzed. Fosfomycin minimum inhibitory concentrations (MICs) were determined using the reference agar dilution method. Resistance was induced by exposing two *K. pneumoniae* strains with a fosfomycin MIC of 4 μg/mL and two *E. coli* strains susceptible to fosfomycin (MIC ≤ 8 μg/mL) to gradually increasing concentrations of the antibiotic. Biofilm-forming capacities, growth rates, and expression levels of selected virulence genes (*fimH* and *papC* in *E. coli*; *entB*, *mrkD*, *uge*, *wabG*, and *ycfM* in *K. pneumoniae*) were compared between variants with low and high fosfomycin MICs.

**Results:**

Of the 78 *E. coli* isolates, 13 (16.6%) were resistant to fosfomycin. Additionally, eight (23.5%) of 34 *K. pneumoniae* isolates exhibited high fosfomycin MICs (MIC > 32 μg/mL). No significant differences in biofilm formation were observed between the variants. However, the expression of the *fimH* gene decreased in one *E. coli* resistant variant compared with its susceptible counterpart. While the expression of the *uge* gene decreased in one *K. pneumoniae* isolate with a high MIC, the expression of the *wabG* gene increased. Slower growth rates were observed in two fosfomycin-resistant *E. coli* strains and one *K. pneumoniae* strain with a high fosfomycin MIC than in their counterparts.

**Conclusion:**

These findings suggest that, in the examined isolates, decreased susceptibility to fosfomycin was associated with slower growth, whereas biofilm formation ability remained largely unaffected. Continued surveillance of fosfomycin resistance is essential owing to its potential implications for bacterial fitness and pathogenicity.

## 1. Introduction

Antibiotic resistance has emerged as one of the most critical public health challenges of the modern era. The excessive and inappropriate use of antibiotics has led to the emergence of infections that are increasingly difficult or even impossible to treat. Infections caused by antibiotic-resistant microorganisms are currently responsible for approximately 700,000 deaths each year. By 2050, it is estimated that antibiotic-resistant infections could cause up to 10 million deaths and impose a global economic burden exceeding 1 trillion USD annually [1]. Fosfomycin has become a preferred therapeutic option for the treatment of uncomplicated urinary tract infections owing to its unique mechanism of action and relatively low rate of resistance development [2,3]. Moreover, the convenience of single-dose administration, which improves patient compliance, further underscores its therapeutic importance. Given these advantages, the use of fosfomycin in treating infections caused by multidrug-resistant (MDR) bacteria is expected to increase. Consequently, the risk of rising fosfomycin resistance rates is anticipated to become more pronounced in the coming years [3]. Fosfomycin exerts its antibacterial effect by inactivating the MurA enzyme, which catalyzes an essential step in bacterial cell wall synthesis, thereby inhibiting peptidoglycan formation. The uptake of fosfomycin into the bacterial cell is mediated by the GlpT and UhpT transport systems. Structural alterations in the MurA enzyme, its overproduction, or changes in the GlpT and UhpT transport systems are major factors contributing to the development of fosfomycin resistance in bacteria [4,5]. The acquisition of fosfomycin-modifying enzymes such as the metalloenzymes FosA, FosB, and FosX or the kinases FomA and FomB also contributes to fosfomycin resistance [6].

The development of antibiotic resistance in bacteria imposes a biological burden known as the fitness cost, which generally results in reduced growth rates and decreased expression of virulence-related genes and proteins [7–10]. However, conflicting results have been reported regarding biofilm formation; some researchers have shown that the fitness cost decreases biofilm formation [11,12], whereas others have reported the opposite effect or no significant difference [9,10,13]. Resistant bacteria must transcribe resistance-related genes in addition to the essential genes required for their metabolic activities. This may lead to a decrease in bacterial growth rate, thereby potentially reducing bacterial virulence and infectivity [14]. Clinically, the fitness cost is important because it influences how long resistant strains can persist once antibiotic pressure is reduced. When the cost is high, the number of resistant strains tends to decline once antibiotic use is discontinued. However, compensatory mutations may emerge to mitigate these costs, allowing resistant strains to remain stable even in the absence of antibiotic pressure [9,15,16]. Thus, a comprehensive understanding of both fitness costs and compensatory adaptive mechanisms is crucial for the rational design of antibiotic rotation and deescalation strategies aimed at effectively managing the long-term dynamics of antimicrobial resistance in clinical settings.

Although fosfomycin is widely used, the development of resistance in clinical strains remains relatively rare. This phenomenon is thought to result from the biological burden (fitness cost) that resistance imposes on bacteria. The unique mechanism of action of fosfomycin makes the development of cross-resistance unlikely [5]. Fosfomycin is primarily recommended for the treatment of uncomplicated urinary tract infections, including cystitis caused by antimicrobial-resistant or extended-spectrum β-lactamase (ESBL)-producing *E. coli*. It also serves as an alternative treatment option for prostatitis caused by ESBL-producing *E. coli* [17]. The Infectious Diseases Society of America does not recommend fosfomycin for the treatment of infections caused by *K. pneumoniae* isolates, which frequently carry fosA or other fosfomycin-hydrolyzing enzymes [18]. However, some studies have reported that multidrug-resistant and NDM-producing clinical *K. pneumoniae* isolates remain susceptible to fosfomycin [19,20]. Furthermore, fosfomycin has the potential to serve as a component of combination therapy in the treatment of infections caused by Enterobacterales.

To date, numerous studies have investigated fosfomycin resistance and its effects on virulence factors such as biofilm formation [21–23]. Nevertheless, our understanding of the effects of fosfomycin resistance on fitness, biofilm formation, and virulence-related genes in clinical *K. pneumoniae* strains remains limited. In this study, we aimed to investigate the potential biological burden (fitness cost) associated with fosfomycin resistance in *E. coli* and *K. pneumoniae* strains.

## 2. Materials and methods

### 2.1. Bacterial strains

A total of 112 bacterial isolates, including 78 *E. coli* and 34 *K. pneumoniae* strains obtained from urine samples collected from various departments at Ege University Faculty of Medicine Hospital, were examined in this study. Bacterial strains were identified using the MALDI-TOF MS system (Biotyper; Bruker Daltonics GmbH and Co. KG, Bremen, Germany), and antibiotic susceptibility testing was performed using the VITEK-2 automated system (bioMérieux SA, Marcy-l’Étoile, France). *E. coli* ATCC 25922 was used as the quality control strain. All bacterial strains were stored at −80 °C in 10% glycerol–brain heart infusion broth until use.

### 2.2. Determination of fosfomycin resistance

The minimum inhibitory concentrations (MICs) of fosfomycin against the collected *E. coli* and *K. pneumoniae* isolates were determined using the reference agar dilution method in accordance with the guidelines of the European Committee on Antimicrobial Susceptibility Testing (EUCAST). Agar plates containing nine different concentrations of fosfomycin (0.5–128 μg/mL) supplemented with a fixed concentration (25 μg/mL) of glucose-6-phosphate were prepared. The lowest concentration of fosfomycin that inhibited visible bacterial growth after 16–18 h of incubation at 37 °C was recorded as the MIC. While the current EUCAST breakpoint (8 μg/mL) was used for interpreting the results of *E. coli* isolates, no accepted breakpoint exists for *K. pneumoniae* isolates in the current EUCAST documents. Therefore, *K. pneumoniae* isolates were not classified as resistant or susceptible. Instead, strains with MIC > 32 μg/mL were described as having a “high fosfomycin MIC” and those with MIC ≤ 32 μg/mL as having a “low fosfomycin MIC”.

### 2.3. Induction of fosfomycin resistance by serial passaging

To investigate the potential effects of fosfomycin resistance on bacterial fitness and virulence factors, two *K. pneumoniae* strains with low fosfomycin MICs (no. 4 and 24) and two fosfomycin-susceptible *E. coli* strains (no. 27 and ATCC 25922) were selected. Among the *K. pneumoniae* isolates, strain no. 4 was resistant to seven antibiotics—nitrofurantoin, amoxicillin/clavulanic acid, imipenem, gentamicin, ciprofloxacin, trimethoprim/sulfamethoxazole, and cefoxitin—whereas strain no. 24 was resistant only to nitrofurantoin. The *E. coli* strain (no. 27) exhibited resistance to four antibiotics—amoxicillin/clavulanic acid, gentamicin, ciprofloxacin, and trimethoprim/sulfamethoxazole. Fosfomycin resistance was induced through serial passaging following the method described by Li et al., with minor modifications [24]. Bacterial suspensions and culture media were prepared as described in the agar dilution method. Each strain was inoculated into culture media containing fosfomycin beginning at half of the MIC (MIC/2) and at twofold increasing concentrations up to 2048 μg/mL. After incubation at 37 °C for 48 h, bacterial suspensions were adjusted to 0.5 McFarland turbidity in sterile saline, and 50 μL of each suspension was transferred onto plates containing the next fosfomycin concentration. Passaging was continued until no visible bacterial growth was observed after 48 h of incubation. The fosfomycin MICs of the resistant variants growing on media containing the highest fosfomycin concentration were confirmed using the agar dilution method and then preserved in 10% glycerol–brain heart infusion broth at −80 °C. Fosfomycin-resistant variants were designated by adding the letter “R” to their original strain numbers.

### 2.4. Comparison of virulence gene expression levels between fosfomycin-susceptible and -resistant variants

The expression levels of the *fimH* and *papC* genes, encoding adhesion molecules, were compared among *E. coli* variants using quantitative real-time polymerase chain reaction (RT-qPCR) [25,26]. In *K. pneumoniae* variants, the expression levels of the *uge*, *ycfM*, and *wabG* genes associated with capsule biosynthesis, the *mrkD* gene encoding an adhesion molecule, and the *entB* gene encoding siderophore biosynthetic enzymes were also analyzed by RT-qPCR [27]. The *recA* and *infB* genes were used as reference genes for *E. coli* and *K. pneumoniae*, respectively. Total RNA was isolated from bacterial cultures, and complementary DNA (cDNA) was synthesized for RT-qPCR analysis. For each reaction, 2 μL of the obtained cDNA was transferred into 96-well RT-qPCR microplates, followed by the addition of 18 μL of reaction mixture to each well. Amplification was performed using the LightCycler 480 II system (Roche Diagnostics GmbH, Mannheim, Germany) under the following cycling conditions: one cycle at 95 °C for 5 min, followed by 40 cycles of denaturation at 95 °C for 10 s, annealing at 58 °C for 15 s, and extension at 72 °C for 20 s. Relative gene expression was calculated using the delta–delta CT method based on the threshold cycle (CT) values. All experiments were performed in triplicate to ensure reproducibility. A minimum twofold change in expression level between the variants was regarded as biologically significant.

### 2.5. Determination of biofilm-forming capacities of fosfomycin-susceptible and -resistant variants

The biofilm-forming capacities of *E. coli* and *K. pneumoniae* variants were determined using the spectrophotometric crystal violet (CV) staining method [28]. The resistant or high-MIC variants were incubated at 37 °C for 16–18 h on Mueller–Hinton agar (MHA) containing half the MIC concentration of fosfomycin (MIC/2) and 25 μg/mL glucose-6-phosphate. The susceptible or low-MIC variants were maintained under identical conditions on MHA plates without antibiotics. After incubation, several colonies were collected, and bacterial suspensions were prepared in 3 mL of sterile saline adjusted to a turbidity equivalent to 0.5 McFarland standard. Biofilm formation was assessed using the CV method in sterile 96-well flat-bottomed microplates [29]. The absorbance values were measured at 570 nm using a microplate reader (Varioskan Flash; Thermo Fisher Scientific Inc., Waltham, MA, USA). All experiments and measurements were performed in triplicate to ensure reproducibility. Tryptic soy broth was used as the negative control, and *Enterococcus faecalis* ATCC 29212 served as the positive control. The optical density (OD) values with standard deviations (SD) were calculated separately for each strain. To determine the biofilm-forming capacities of the variants, the OD values of each variant were compared with that of the negative control, as described in Table 1 [28,29]. The tested strains and their variants were classified as nonbiofilm, weak (low), moderate, or strong biofilm formers. Statistical analyses were performed using the Student’s t-test in GraphPad Prism 8 (GraphPad Software, San Diego, CA, USA). A p < 0.05 was considered statistically significant.

### 2.6. Determination of growth rates of fosfomycin-susceptible and -resistant variants

The growth rates of *E. coli* and *K. pneumoniae* variants were determined using a spectrophotometric method [30]. For this purpose, several colonies were collected from fosfomycin-resistant or high-MIC variants grown on MHA containing fosfomycin, and from fosfomycin-susceptible or low-MIC variants grown on MHA without fosfomycin. These colonies were used to prepare bacterial suspensions in sterile saline adjusted to a turbidity of 0.5 McFarland standard (approximately 1.5 × 10^8^ colony-forming units/mL). Each suspension (5 μL) was inoculated into tubes containing 5 mL of Luria–Bertani broth. The inoculated tubes were incubated at 37 °C in a shaking incubator at 200 rpm for 10 h to ensure optimal aeration. The time point at which bacteria were inoculated into the broth was designated as time 0 h, and samples were collected at 0, 2, 4, 6, 8, and 10 h of incubation. Each sample (100 μL) was transferred in triplicate into sterile 96-well microplates. The OD was measured at 600 nm using a spectrophotometer (Varioskan Flash; Thermo Fisher Scientific Inc., Waltham, MA, USA), and absorbance values were recorded. Differences in bacterial growth rates were assessed based on the obtained OD values. For each sampling time point, the OD values of resistant and susceptible variants were compared using the Student’s t-test in GraphPad Prism 8 (GraphPad Software, San Diego, CA, USA). A p < 0.05 was considered statistically significant. Statistical significance levels were denoted as p < 0.05*, p < 0.01**, and p < 0.001***.

## 3. Results

### 3.1. Fosfomycin resistance

The MICs of a total of 112 isolates, including 78 *E. coli* and 34 *K. pneumoniae* strains, were determined using the reference agar dilution method. According to EUCAST guidelines, *E. coli* strains with MIC > 8 μg/mL were considered resistant to fosfomycin. For *K. pneumoniae* isolates, those with MIC > 32 μg/mL were designated as having high fosfomycin MICs. Based on these criteria, 13 (16.6%) of 78 *E. coli* isolates were resistant to fosfomycin, whereas eight (23.5%) of 34 *K. pneumoniae* isolates exhibited high fosfomycin MIC values. The distribution of fosfomycin MIC values among the tested strains is presented in Table 2.

### 3.2. Fosfomycin-susceptible and-resistant bacterial variants

Fosfomycin-resistant and high-MIC variants were generated from two fosfomycin-susceptible *E. coli* strains (no. 27 and ATCC 25922) and two *K. pneumoni*ae strains with low fosfomycin MICs (no. 4 and 24). Serial passaging on agar plates containing increasing concentrations of fosfomycin (from MIC/2 to 2048 μg/mL) was continued until no visible growth was observed after 48 h of incubation. The fosfomycin MICs for *K. pneumoniae* variants increased from 4 μg/mL to >2048 μg/mL. The fosfomycin MIC for *E. coli* strain no. 27 increased from <0.5 μg/mL to 1024 μg/mL, while that for the *E. coli* ATCC 25922 reference strain increased from <0.5 μg/mL to 512 μg/mL.

### 3.3. Differences in virulence gene expression levels between fosfomycin-susceptible and-resistant variants

**Figure 1:**
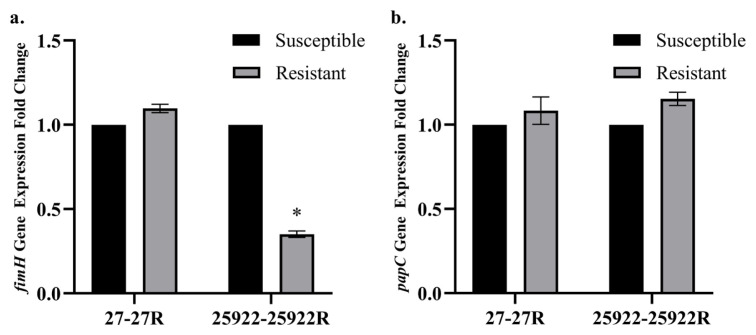
Relative expression levels of the *fimH* (a) and *papC* (b) genes in fosfomycin-resistant and -susceptible *E. coli* variants. * indicates a minimum twofold change in expression level.

A significant decrease in the expression level of the *fimH* gene was observed in the *E. coli* 25922R variant compared to its parental *E. coli* 25922 strain, whereas no significant change was detected between the susceptible and resistant variants of *E. coli* strain no. 27 (Figure 1a). No significant difference was observed in the expression levels of the *papC* gene between fosfomycin-resistant and susceptible *E. coli* variants (Figure 1b).

**Figure 2:**
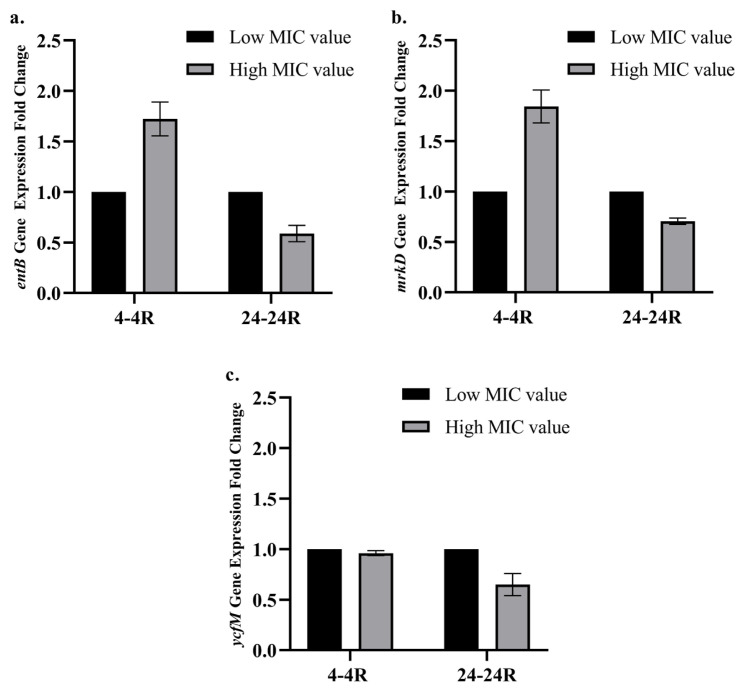
Relative expression levels of the *entB* (a), *mrkD* (b), and *ycfM* (c) genes in *K. pneumoniae* variants. Low fosfomycin MIC value: 4 μg/mL; high fosfomycin MIC value: 2048 μg/mL. * indicates a minimum twofold change in expression level (not observed in this figure).

**Figure 3:**
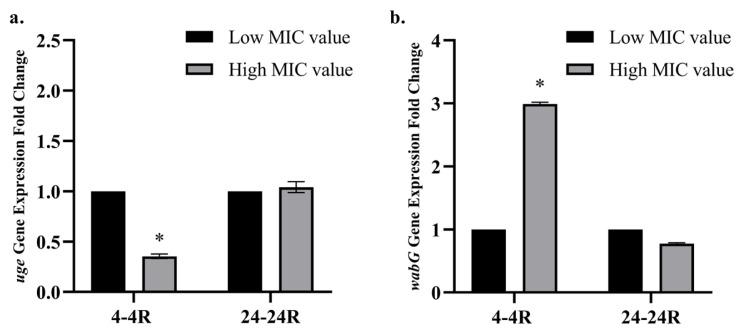
Relative expression levels of the *uge* (a) and *wabG* (b) genes in *K. pneumoniae* variants. Low fosfomycin MIC value: 4 μg/mL, high fosfomycin MIC value: 2048 μg/mL. * indicates a minimum twofold change in expression level.

The expression levels of the *entB* (Figure 2a), *mrkD* (Figure 2b), and *ycfM* (Figure 2c) genes did not differ significantly among the *K. pneumoniae* variants. A significant decrease in the expression of the *uge* gene was observed in the *K. pneumoniae* 4R variant compared to its parental 4 variant. Conversely, no significant difference in the expression level of the same gene was detected between *K. pneumoniae* 24 and 24R variants (Figure 3a). Additionally, the *wabG* gene was significantly overexpressed in the *K. pneumoniae* 4R variant compared with its parental 4 variant. In contrast, no significant difference in the expression level of the *wabG* gene was observed between *K. pneumoniae* 24 and 24R variants (Figure 3b).

### 3.4. Biofilm-forming capacities of fosfomycin-susceptible and -resistant variants

**Figure 4:**
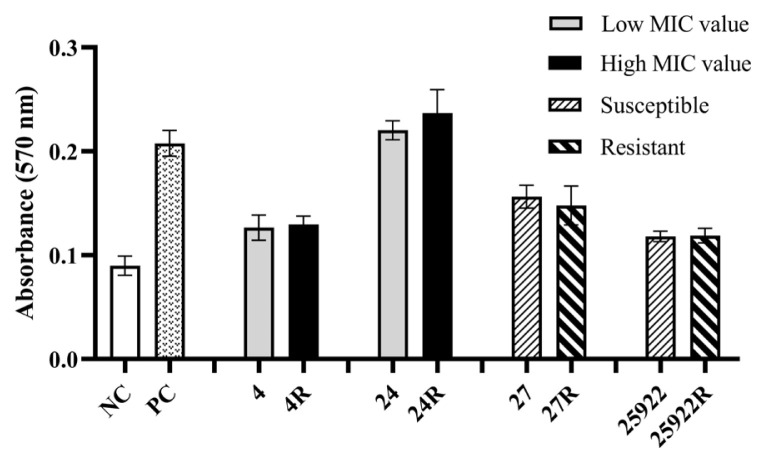
Optical density (OD_570_) values of the examined *E. coli* and *K. pneumoniae* variants. Low fosfomycin MIC value: 4 μg/mL, high fosfomycin MIC value: 2048 μg/mL. NC: negative control; PC: positive control.

The tested strains and their variants were classified as nonbiofilm, weak (low), moderate, or strong biofilm formers according to the CV staining method. It was observed that *K. pneumoniae* strain no. 4 and its resistant variant (4R), as well as the two *E. coli* strains (no. 27 and ATCC 25922) and their resistant variants (27R and 25922R), were classified as weak biofilm formers. Moreover, *K. pneumoniae* strain no. 24 and its resistant variant (24R) exhibited a moderate level of biofilm formation. No significant difference was detected in the biofilm-forming capacities between the *E. coli* and *K. pneumoniae* variants examined (Figure 4).

### 3.5. Comparison of growth rates between fosfomycin-susceptible and -resistant variants

**Figure 5:**
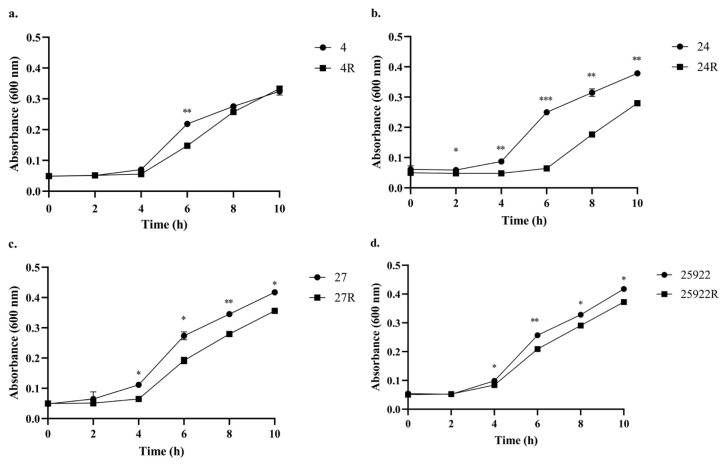
Growth curves showing the absorbance (OD_600_) values of the examined *E. coli* and *K. pneumoniae* variants labeled as 4/4R (a), 24/24R (b), 27/27R (c), 25922/25922R (d) over time. * p < 0.05, ** p < 0.01, *** p < 0.001.

The growth rates of the variants were determined by measuring the OD of samples collected at fixed time intervals from the bacterial suspensions. *K. pneumoniae* strain no. 4 exhibited a significantly higher absorbance value only at the 6th h compared with its resistant variant (4R). No significant differences were observed in the absorbance values between the variants of the same strain at the other time points (Figure 5a). However, *K. pneumoniae* strain no. 24 exhibited significantly higher absorbance values than its resistant variant (24R) at 2, 4, 6, 8, and 10 h (Figure 5b). Similarly, at 4, 6, 8, and 10 h, *E. coli* strains no. 27 and ATCC 25922 exhibited higher absorbance values than their resistant variants (27R and 25922R) (Figures 5c and 5d).

## 4. Discussion

Because of the prolonged discovery process and the high costs associated with developing new antibiotics, the therapeutic options available for treating antibiotic-resistant bacteria are steadily decreasing [31]. Consequently, there has been renewed interest in so-called “old” antibiotics, such as colistin and fosfomycin [32]. Since its discovery, fosfomycin has been used primarily for the treatment of uncomplicated urinary tract infections, with limited application to other types of infections [5,33]. Given the limited number of antibiotics available for treating infections caused by antibiotic-resistant bacteria and its proven effectiveness against ESBL-producing strains, fosfomycin is increasingly being considered for therapeutic use beyond urinary tract infections. Furthermore, several studies have demonstrated that fosfomycin is effective against MDR *K. pneumoniae* isolates, thereby expanding its potential clinical applications [19,20]. However, the expanded clinical use of fosfomycin may accelerate the emergence of resistance [34,35]. In the present study, the MICs of fosfomycin were determined using the reference agar dilution method. Accordingly, the resistance rate to fosfomycin was 16.6% (13/78) among *E. coli* isolates. In a study conducted by Soydan et al. (2021), fosfomycin resistance was reported in 4.9% (4/89) of *E. coli* isolates [36]. In a 2022 study by Öcal and colleagues, fosfomycin resistance was detected in only six (3%) of 175 *E. coli* strains using the agar dilution method (MIC > 32 μg/mL indicating resistance) [37]. The fosfomycin resistance rate (approximately 17%) observed among *E. coli* isolates in this study was higher than those reported in previous studies. However, this discrepancy may be explained by the updated EUCAST breakpoints for fosfomycin, which lowered the susceptibility threshold for *E. coli* from 32 μg/mL to 8 μg/mL. Globally, numerous studies have addressed this issue; however, some did not employ the reference agar dilution method, and in certain cases, fosfomycin resistance was interpreted according to the Clinical and Laboratory Standards Institute breakpoints [38–41].

In this study, fosfomycin-resistant variants were developed from two fosfomycin-susceptible *E. coli* and two *K. pneumoniae* strains, and the expression levels of several virulence-associated genes were analyzed in these variants. The underlying mechanisms of fosfomycin resistance were not investigated in these selected strains, which represents a limitation of the present study. The analysis focused on variations in the *fimH* and *papC* genes in *E. coli* strains, and in the *entB*, *mrkD*, *uge*, *ycfM*, and *wabG* genes in *K. pneumoniae* strains. The *fimH* gene, responsible for adhesion to host epithelial cells, has been reported as one of the most frequently detected virulence factors in uropathogenic *E. coli* isolates. Its prevalence has been reported to range from 68% to 100% in various studies [25,42–45]. The pyelonephritis-associated pili (*pap*), also known as P fimbriae, are adhesins found in *E. coli* strains associated with pyelonephritis and significantly contribute to the virulence of uropathogenic *E. coli* [46]. In this study, the expression level of the *fimH* gene did not differ significantly between *E. coli* variants 27 and 27R. However, its expression was significantly reduced in the fosfomycin-resistant *E. coli* 25922R variant compared with the susceptible *E. coli* 25922 strain. No significant difference was observed in the expression levels of the *papC* gene between fosfomycin-resistant and susceptible *E. coli* variants. Harwalkar and colleagues reported that the *papC* gene was detected in 72.9% of ciprofloxacin-susceptible uropathogenic *E. coli* strains, whereas it was present in only 40.2% of ciprofloxacin-resistant strains [47]. Moreover, Pira et al. demonstrated that fosfomycin-resistant *E. coli* strains exhibited reduced adhesive capacity [48]. These findings suggest that resistant bacteria may prioritize other essential metabolic processes over the synthesis of P fimbriae, thereby reallocating their cellular energy and resources. Campos et al. reported that several resistance-associated genes were significantly downregulated or upregulated in fosfomycin-resistant *E. coli* subpopulations compared with their parental heteroresistant isolates [22]. However, they also reported that these alterations in gene expression did not impose a detectable fitness cost. Bermudez et al. observed that fosfomycin resistance did not impair virulence in uropathogenic *E. coli* strains [21]. Collectively, these studies suggest that fosfomycin-resistant isolates may display transcriptional alterations in virulence-related genes, although such molecular changes are not always manifested phenotypically.

*K. pneumoniae* regulates capsule and polysaccharide biosynthesis through genes such as *uge*, *wabG*, and *ycfM*. The absence of these genes impairs capsule formation in the bacteria, resulting in a marked reduction in virulence [49,50]. Some genes, including *fimH* and *ycfM*, are responsible for the synthesis of adhesion molecules that facilitate bacterial attachment to host epithelial cells [51]. Enterobactin, encoded by the *entB* gene, is one of the major siderophores produced by *K. pneumoniae* [52]. In this study, no significant differences were observed in the expression levels of the *entB*, *mrkD*, and *ycfM* genes between the high- and low-MIC *K. pneumoniae* variants. However, in the high-MIC *K. pneumoniae* variant 4R, the expression of the *uge* gene was significantly reduced, whereas the *wabG* gene was upregulated compared with its parental 4 variant. In contrast, no significant differences in the expression levels of these genes were observed between *K. pneumoniae* 24 and 24R variants. Since both the *uge* and *wabG* genes are involved in bacterial polysaccharide biosynthesis, it is plausible that the decreased expression of *uge* in the *K. pneumoniae* 4R variant was compensated by the overexpression of *wabG*. This study presents, for the first time, data demonstrating how fosfomycin resistance influences the expression of virulence-associated genes in clinical *K. pneumoniae* isolates. Although fosfomycin resistance did not induce alterations in most of the virulence-associated genes examined in *K. pneumoniae* variants, compensatory regulation may occur among genes with overlapping functions.

Biofilm formation plays a crucial role in *Enterobacteriaceae* infections and is frequently observed among *K. pneumoniae* isolates [53,54]. Consistent with our biofilm formation results, no significant differences were detected between the *E. coli* and *K. pneumoniae* variants examined. These findings suggest that the acquisition of fosfomycin resistance did not alter the biofilm-forming capacity of the strains examined.

The development of urinary tract infections requires the presence of a sufficient bacterial load within the urinary tract, and these bacteria must possess the ability to adhere to host tissues. The continuous flushing of the urinary tract by urine creates a challenging environment for bacterial adherence. Moreover, the biological disadvantage associated with the acquisition of antibiotic resistance reduces bacterial growth rates, thereby hindering the attainment of the critical inoculum required for infection establishment. The observation that fosfomycin resistance can be easily acquired in vitro, whereas clinical isolates from infections often remain susceptible to fosfomycin, supports this hypothesis [55]. In the present study, the growth rates of fosfomycin-susceptible and resistant bacterial variants were determined, and the results indicated that fosfomycin resistance generally tended to reduce the growth rates of the examined strains, except for the *K. pneumoniae* variant 4R. Pira et al. compared the growth rates of fosfomycin-resistant *E. coli* (two strains) and *K. pneumoniae* (one strain) with those of their isogenic susceptible counterparts. They found that the growth rates of resistant *E. coli* variants were lower than those of the susceptible variants. However, no significant difference in growth rate was observed between the *K. pneumoniae* variants [48]. Additionally, other studies have suggested that the acquisition of resistance to certain antibiotics may not impose a measurable fitness cost among members of the order Enterobacterales [56].

The results of this study revealed that, although some differences were detected in the expression of virulence-associated genes between fosfomycin-susceptible and -resistant *E. coli* variants, as well as between low- and high-MIC *K. pneumoniae* variants, these differences were not reflected in their biofilm-forming capacities. Furthermore, it was determined that fosfomycin resistance generally imposes a fitness cost on the examined strains. Although the inclusion of only four strains represents a limitation of this study, it nevertheless provides valuable insights into the biological profiles of the selected clinical isolates.

In light of the increasing rates of antibiotic resistance, expanding the use of fosfomycin against MDR pathogens, including *K. pneumoniae* isolates, is being considered. In this context, the expression levels of virulence-associated genes in fosfomycin-susceptible and -resistant *K. pneumoniae* variants were assessed for the first time in this study. The findings of this study may contribute to the rational evaluation of fosfomycin use in *K. pneumoniae* infections. These findings also suggest that the clinical use of fosfomycin should be carefully monitored to prevent the emergence of resistance.

## Figures and Tables

**Table 1:** Classification of biofilm-forming capacities of the examined strains.

OD ≤ Negative control (NC)	No biofilm formation (−)
NC < OD ≤ 2 × NC	Low biofilm formation (+)
2 × NC < OD < 4 × NC	Moderate biofilm formation (++)
4 × NC ≤ OD	Strong biofilm formation (+++)

**Table 2:** Fosfomycin MICs of *E. coli* and *K. pneumoniae* strains determined by the agar dilution method. Underlined values indicate the number of fosfomycin-resistant isolates.

	Fosfomycin minimum inhibitory concentrations (MICs) (μg/mL)								
	<0.5	1	2	4	8	16	32	64	128
***Escherichia coli*** ** (n = 78)**	40	19	2	2	2	**4**	**2**	**6**	**1**
***Klebsiella pneumoniae*** ** (n = 34)**	8	1	3	6	3	2	3	0	8

## Data Availability

All data generated or analyzed during this study are included in this published article.
